# The Requirement of Genetic Diagnostic Technologies for Environmental Surveillance of Antimicrobial Resistance

**DOI:** 10.3390/s21196625

**Published:** 2021-10-05

**Authors:** Karine Caron, Pascal Craw, Mark B. Richardson, Levente Bodrossy, Nicolas H. Voelcker, Helmut Thissen, Tara D. Sutherland

**Affiliations:** 1 CSIRO Health & Biosecurity, Canberra, ACT 2602, Australia; karine.caron@csiro.au; 2 CSIRO Oceans & Atmosphere, Hobart, TAS 7004, Australia; pascal.craw@csiro.au (P.C.); lev.bodrossy@csiro.au (L.B.); 3 CSIRO Manufacturing, Clayton, VIC 3168, Australia; mark.richardson@csiro.au (M.B.R.); nicolas.voelcker@monash.edu (N.H.V.); helmut.thissen@csiro.au (H.T.); 4 Melbourne Centre for Nanofabrication, Victorian Node of the Australian National Fabrication Facility, Clayton, VIC 3168, Australia; 5 Monash Institute of Pharmaceutical Sciences, Monash University, Parkville, VIC 3052, Australia

**Keywords:** antimicrobial resistance, AMR, one health, diagnostics, biosensing, ARG, environmental monitoring

## Abstract

Antimicrobial resistance (AMR) is threatening modern medicine. While the primary cost of AMR is paid in the healthcare domain, the agricultural and environmental domains are also reservoirs of resistant microorganisms and hence perpetual sources of AMR infections in humans. Consequently, the World Health Organisation and other international agencies are calling for surveillance of AMR in all three domains to guide intervention and risk reduction strategies. Technologies for detecting AMR that have been developed for healthcare settings are not immediately transferable to environmental and agricultural settings, and limited dialogue between the domains has hampered opportunities for cross-fertilisation to develop modified or new technologies. In this feature, we discuss the limitations of currently available AMR sensing technologies used in the clinic for sensing in other environments, and what is required to overcome these limitations.

## 1. Introduction

Antimicrobial resistance is recognised as the most urgent emerging threat to human and animal health today [1,2,3]. A highly publicised economic report has projected that deaths from infections previously treatable with antibiotics will rise exponentially from 700,000/yr (2015) to 10 million/yr by 2050 [4]. The impact of AMR on livestock in the same period is predicted to result in up to a 7.5% global decrease in productivity [5]. The World Bank has projected a broad range of AMR-associated declines in development indicators, including a decline in global GDP of between 3.8–5% by 2050, an increase in 28.3 million people living in extreme poverty, a 1.1% contraction in global real exports, and ballooning global healthcare cost from $300 billion to >$1 trillion per year [5].

The World Organisation for Animal Health, the Food and Agriculture Organization of the United Nations, and World Health Organisation have each conceded that “resistance anywhere is resistance everywhere” [6], and that addressing the rising threat of AMR requires an inclusive, integrated, and multisectoral One Health approach recognising the interconnectivity between human health, agriculture and the environment. Here, surveillance systems are particularly critical components [7]. Systematic AMR surveillance is currently used to varying degrees in the healthcare systems of most developed countries, while surveillance in food, wildlife, and domestic animals is also performed regularly in some countries [e.g., the Norwegian Surveillance System for Antimicrobial Drug Resistance (NORM and NORM-Vet), the Danish Integrated Antimicrobial Resistance Monitoring Program (DANMAP), National Antimicrobial Resistance Monitoring System in the United States (NARMS); Swedish Veterinary Antimicrobial Resistance Monitoring (SVARM)]. However, equivalent surveillance systems for the environment are in their infancy. GLASS, a global antimicrobial resistance surveillance system supported by WHO, OIE and FAO, is currently assessing the usefulness of environmental surveillance approaches to identify which are most appropriate, with a focus on metagenomics and yearly surveillance of the frequency of extended spectrum beta-lactamase (ESBL) *E. coli* in hospitalised patients, the food chain, and the environment [8].

**Figure 1:**
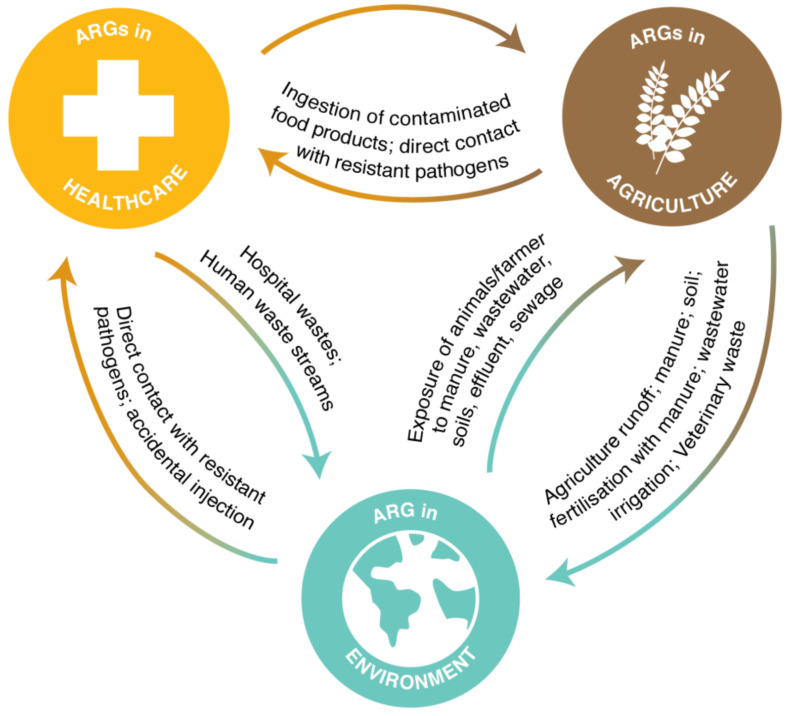
Commonly cited AMR transmission routes for antibiotic resistance genes (ARGs) between healthcare, agricultural and environmental domains.

A key objective of environmental AMR surveillance systems is to guide interventions that reduce the spread of AMR from the environment to the clinic or to agricultural production systems and vice versa. Identifying pathways of transmission, and the risks associated with these pathways, requires an understanding of the AMR load in the environment, what constitutes an AMR risk, and how this risk changes in response to natural variations or human interventions [4,9]. Commonly cited transmission routes between healthcare, agricultural and environmental domains are shown in Figure 1. Among the prevailing modes of transmission is the diversion of faecal microbes via waste streams into the environment (Figure 1). Consequently, the environment is a known reservoir of resistant bacteria and antibiotic resistance genes (ARGs). Little is currently known about the risk and likelihood of transmission back from the environment to the healthcare and agricultural domains.

In the healthcare domain, antimicrobial agents used in treatment or clinical sanitization regimens create evolutionary pressure on microbial populations, selecting drug-resistant strains [10]. Outside this domain, the narrative is less obvious. The major drivers of AMR within the environment are partially obscured by the sub-minimal inhibitory concentrations of antimicrobial agents that are far from sufficient to explain the observed prevalence of AMR [11]. The effect of sub-lethal levels of antibiotics [10], antibiotic structural analogues, and the presence of metals and biocides derived from pharmaceutical and agricultural industries are all receiving due attention [12]. The factors contributing to the transit of ARGs between environmental and agricultural locales, as well as healthcare settings, are similarly ill defined [13]. The establishment of quantitative environmental surveillance systems based on relevant AMR biomarkers will be an important future asset in building predictive models for AMR prevalence and ARG transfer between the environment and healthcare/agricultural domains.

Currently, surveillance of AMR is primarily based on data obtained from the clinic to guide treatment of infections in a patient [14]. The use of this data for surveillance is a secondary purpose and diagnostic methods have been designed to suit the clinical need rather than the requirements of surveillance. Clinical diagnostics are commonplace and primarily based on phenotypic Antibiotic Susceptibility Testing (AST) to identify what antibiotics could be used to treat an infection, coupled to methods that identify the host organism. Such phenotypic monitoring generally requires the isolation and identification of individual species, growth monitoring of individual cells, or other adjustments to account for differences in growth rates between bacterial species. The primary objectives of clinical diagnostic tests are quite distinct from that of environmental surveillance and it stands to reason that diagnostics developed to guide patient treatment in the clinic may not be translatable to environmental AMR surveillance.

Outside of the clinic, AST in key indicator species is used in some surveillance systems to monitor AMR in wildlife, domestic animals, and food (for example, NORM and NORM-Vet; DANMAP; NARMS; SVARM). Phenotypic tests for ESBL *E. coli* from environmental samples have been proposed as global One Health monitoring systems suitable for all countries, regardless of their scientific infrastructure, based on predictions from the current state of AMR that these organisms will be responsible for the greatest AMR economic burden. However, phenotypic monitoring is not generalizable as only around 2% of species can be cultured. In order to monitor the emergence of AMR in new areas, guide interventions, or identify AMR hotspots, more broadly applicable techniques are imperative for AMR monitoring in the environment [11].

The alternative to phenotypic testing is genetic testing—identifying DNA sequences that act as biomarkers of AMR potential, possibly ARGs or mobile genetic elements associated with ARGs such as class 1 integron/integrase sequences. These methods can be quantitative, are species agnostic, and do not rely on the culture of bacteria. Metagenomics and whole genome sequencing can also provide information regarding genetic context of the DNA biomarker, i.e., the pathogenicity of the host organism or association of the ARG with mobile genetic elements that may promote transfer of the ARG from one species to another [13]. Knowledge of this genetic context provides important information in regard to the potential risk that the associated ARG provides. Genetic methods are limited to surveillance of known biomarkers. However, data sets collected from methods such as metagenomics or whole genome sequencing can be analyzed retrospectively to identify biomarkers as they become validated, or interrogated to identify predicted ARGs that then can be validated in the laboratory. A second shortcoming of genetic methods and the primary reason why these methods are less popular in the clinic, lies in the fact that detecting and/or quantifying an ARG in a sample does not necessarily correlate with a resistant phenotype within that sample. To overcome this, much research effort is being invested into predicting antibiotic susceptibility from genome data alone [12].

Some methods, such as metagenomic and whole genome sequencing are currently and increasingly being used to obtain a broad picture of antibiotic resistance across a wide range of organisms [6,9,15]. However, the cost, complexity and need for skilled operators of these methods limits their use to small sample numbers and precludes their adoption in on-going, large-scale environmental surveillance especially in resource limited settings. Therefore, from an economic standpoint, whilst whole-genome and metagenomic methods are potentially suitable as nodes in a broader surveillance network, alternatives must be considered to expand the network to collect data regularly and across all environments.

It is clear that the use of AMR surveillance data to understand and influence the exchange of AMR between the environment, human and animal healthcare settings requires knowledge of the most pertinent biomarkers. However, such knowledge is not currently available. Therefore, in this perspective we focus on identifying suitable technologies for achieving this vision, under the premise that specific ARG targets will assuredly be the focus of future review articles.

In the first instance, we critically assess the concentrations at which ARGs are found in the environment to determine the sensitivity required for surveillance. Further to the required sensitivity, the selectivity of potential AMR surveillance technologies is also discussed with reference to the quotient of target and non-target nucleic acid encountered in the environment. These parameters are then used to establish thresholds and restrict the choices related to suitable candidate technologies, which are discussed in greater detail. Finally, options for sample collection are summarized.

## 2. Sensitivity and Selectivity Requirements for Environmental AMR Surveillance

The level of individual ARGs in different environments is highly variable with recent studies on water samples reporting ARGs copy numbers up to 10^8^/mL [16,17,18], and in sediment of 10^11^/g [16,17,18,19]. A summary of earlier studies [20] reported levels up to 10^12^/g livestock waste, 10^8^/g hospital waste and 10^7^/g municipal waste. In addition to highly variable copy numbers, environmental DNA complexity is also highly variable: Dong et al. [19] found 10^3^–10^10^ ARGs within 9–120 μg DNA/g dry sample, which equates to around 1–10^7^ ARG nucleotides/10^11^ total nucleotides. In intracellular DNA, the equivalent value measured was 1–10^7^ ARG nucleotides/10^10^ total nucleotides. Whilst there is value in understanding what ARGs are present in an environmental sample, guidance on the success of interventions to manage AMR requires quantifying the level of ARG, or AMR biomarker, within these highly variable and complex samples to, for example, compare levels before and after intervention.

We are not aware of any quantitative DNA detection method that has a limit of detection and range to match the levels of ARGs across all environments. Almost always results reported for limit of detection and range for DNA detection technology are obtained under ideal laboratory conditions and it is reasonable to assume a significant reduction in sensitivity when analyzing complex samples under environmental conditions. Even assuming the ability of achieving 10 or 100-fold sample concentration and optimal DNA extraction, there is currently no detection technique that can detect DNA across the 0–10^12^ dynamic range observed for ARG sequence abundance in environmental samples. The gold standard commercial method for DNA quantification is quantitative polymerase chain reaction (qPCR) which has a dynamic range of 10–10^7^ copies/reaction in real applications [21,22], with a theoretical limit of detection of 3 copies/reaction [23]. Consequently, for any generic environmental DNA detection system, there will be a requirement to concentrate and dilute samples to cover the dynamic range of any detection method prior to detection.

**Figure 2:**
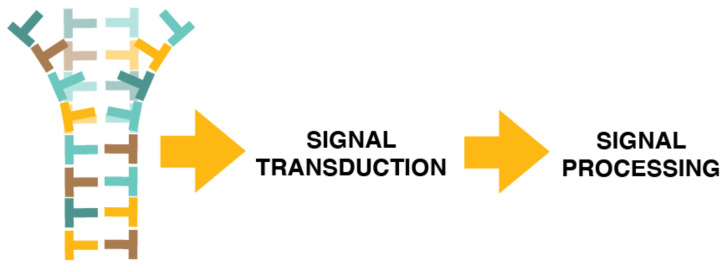
A simplistic illustration of the three components required in DNA-DNA hybridisation diagnostics: hybridisation of the target sequence to a DNA probe, a method to recognise that hybridisation, commonly referred to as the signal transduction method, and processing of that signal into a form compatible with end user requirement. Two of these components, the kinetics of DNA hybridization and the signal transduction method used to detect this hybridisation, determine the sensitivity of the method.

This raises the question of what methods may be suitable to quantify ARGs from environmental samples? The sensitivity and specificity of any DNA detection system results from a combination of (a) the sensitivity and selectivity of the DNA binding event and (b) the sensitivity of the signal transduction mechanism (see Figure 2) [24].

Although DNA sequences can be recognised by molecules that contain specific DNA binding sites such as antibodies or molecular imprinted polymers, undoubtedly the most sensitive and selective methods to detect DNA are based on DNA hybridisation. For example, a 24 nucleotide DNA strands bind complementary strands with attomolar binding affinity compared to nanomolar binding to monoclonal antibodies [25]. Consequently, given the requirements for environmental sensing we focus on methods based on DNA-DNA hybridization.

### Maximising Sensitivity of DNA-DNA Hybridisation

The sensitivity of the signal transduction component is generally the focus of attention in DNA sensing technology development, with the DNA-DNA hybridisation component often overlooked. Existing and emerging DNA sensing technology has a bias towards detection based on relatively short regions of hybridisation, most likely as a result of the ease of which short DNA sequences can be purchased commercially. Sensitivity and selectivity of DNA-DNA hybridisation is dependent on the length of the hybridising probe, as the binding and dissociation kinetics of DNA duplex formation depends on the length of complementarity between the strands. As an example, binding of 8 nucleotide sequences to complementary targets is 3 times faster than that of a 12 nucleotide sequence [26], but more importantly the dissociation of the longer sequence is over 40 times slower, leading to an overall binding affinity that is 14 times tighter, despite the longer sequence only having an extra 4 nucleotides [26]. In a complex mixture, use of longer probes with lower DNA-DNA dissociation rates leads to increased sensitivity. Experimentally, this has been demonstrated in DNA arrays where the ability of <30 nucleotides probes to hybridize target sequences is limited to situations when the target represents at least 1% of total DNA [27]. In contrast, a 70 nucleotide probe can detect a specific gene within a complexity of 100 bacterial genomes (equalling approximately 500 M nucleotides) [28]. In order to achieve the maximum sensitivity, hybridization-based diagnostic approaches for environmental AMR surveillance should consider regions of homology between a probe and a target of at least 50 nucleotides (with the optimal length for any detection method to be determined experimentally).

A criticism of methods utilising hybridization of longer DNA sequences is that they may be limited by cross-hybridization of non-target sequences, normally cited as sequences with greater than 75% similarity. Whilst this is a genuine concern it is circumnavigated by designing probes that do not cross-react with non-target sequences. In many cases this is a trivial process—when 100 base pair fragments of the *E. coli’s* β-lactamase gene, EC_3.5.2.6, are compared to all gene sequences available within the NCBI DNA database using the BLAST algorithm, no nonhomologous sequences are identified and all sequences with >65% identity were found to correspond to a β-lactamase homolog.

There are methods that can specifically identify DNA sequences with high sensitivity such as DNA endonuclease-targeted CRISPR trans reporter [29]. However, these techniques may have limited utility in environmental sensing because there is substantial sequence diversity among ARGs. Consequently, methods that identify specific DNA sequences but not homologs have limited applicability in this context. To understand the level of ARG biomarkers in the environment, the surveillance method must be able to accommodate the known sequence diversity within ARG families.

## 3. Sensing Methods That May Meet the Requirements for an Environmental Surveillance System

### 3.1. Nucleic Acid Amplification-Based Methods

These methods are based on detection of target DNA by, most commonly, 18–30 nucleotide complementary DNA primers, followed by cycles of enzymatic DNA synthesis from the primer-target DNA. Accumulation of labelled target copies are normally detected optically. The use of repeated sequence specific hybridization of two distinct primers coupled with high-fidelity exponential replication of labelled target sequences makes these methods highly popular due to both their sensitivity and accuracy. As the target is amplified, the signal transduction label is amplified and the ultimate sensitivity of these techniques is not limited by the signal transduction label. Instead, sensitivity is primarily determined by the initial DNA-DNA hybridisation reaction, which, assuming optimised reaction conditions, will be influenced by the length and GC content of the probe and the presence of secondary structure in the target or probe. The typical reaction time of nucleotide acid amplification based methods is 1–2 h.

Polymerase chain reaction (PCR) was the first of these methods and this technique has been used extensively in clinical diagnostics and more recently in AMR sensing studies in freshwater [30,31,32], drinking water [33,34], marine waters [35,36] and waste water [37,38,39]. Whilst in situ and autonomous instrumentation for AMR monitoring in the environment are only emerging, a few instruments for aquatic monitoring that use PCR for in situ sensing have been demonstrated [40,41]. Recent advances in PCR hardware, particularly in heat transfer, have reduced cycling times to as little as 2 min on commercially available instruments (BV, 2016) [42], a feature which will be appealing in situations where power consumption and/or rapid notification is a priority.

In addition to PCR, isothermal nucleic acid amplification methods have been developed for DNA amplification. These methods employ enzymatic or biochemical methods rather than energy intensive thermal cycling to drive the amplification process and hence are particularly well suited for point-of-use devices, resource limited settings and in situ sensing for AMR monitoring in the environment. A number of these methods have been demonstrated for AMR detection in clinical applications [43].

As assays for AMR migrate into the field for point-of-use testing and in situ instrumentation, the sensitivity, specificity, simplicity and tolerance to varied sample types make DNA amplification-based assays an obvious lead candidate technology. However, while offering the gold standard in sensitivity and selectivity, these techniques rely on temperature-sensitive reagents, especially enzymes, that can degrade rapidly when stored incorrectly. Multiplex assays [30,44,45] and high throughput PCR for AMR targets [46] have emerged to perform multiple reactions in parallel, broadening the number of targets. However, to reach their full potential in sensitivity and selectivity, the reaction conditions need to be optimized for each sequence/primer set, which limits the sensitivity/selectivity that can be reached when reactions are multiplexed. Furthermore, the presence of environmental contaminants in target DNA samples has varying detrimental effects on all these methods. Caution is also required in experimental design as commercial samples of enzymes can contain 100–10,000 stoichiometric excess of bacterial DNA, often with high concentration of ARGs originating from bacterial production systems. These contamination issues were a prominent topic of discussion in the early 1990s [47,48,49,50], and can obscure DNA analyses to the present day [51].

### 3.2. Surface Immobilized DNA Technologies

Technologies where DNA sequences complementary to target sequences are immobilized on a surface may also be promising candidates in the context of AMR monitoring. The capacity to bind target DNA by hybridisation to the immobilized DNA, then wash away contaminants is a significant advantage of this technology.

Traditionally, detection comes after sample DNA is labelled and hybridization events measured by quantifying amounts of the bound label. Using such DNA methods, detection of ARGs in bacteria has been amply demonstrated [52]. However, procedures to label the DNA sample are costly, time consuming, and require skilled technicians and hence are not suited to automated or point-of care-detection. More viable alternatives to quantifying hybridization for automated or point-of care-detection rely on methods that detect surface changes post hybridization. For example, surface plasmon resonance, calorimetry, quartz crystal microbalance measurements, biolayer interferometry, reflectometric interference spectroscopy [53], or electrochemistry [54,55]. These methods do not rely on labile chemicals or reagents and all have been miniaturized and are regarded as being low cost options for diagnostic devices [53,56,57,58]. Most have been developed for real-time or close to real-time monitoring but they have not been trialed for surveillance of environmental DNA. Generally, these signal transduction techniques require significantly greater amounts of target DNA than DNA amplification techniques in order to generate measurable signal, a limitation that may be overcome by using the DNA probes as capture agents over a longer time period. Based on the discussion above, coupling probes of greater than 50 nucleotide sequences to capture target sequences with these surface-based detection methods on large sample sizes may provide a method to automate detection of AMR biomarkers from environmental DNA samples, most likely in situations where target DNA is more abundant.

However, the use of large sample volumes complicates device design based on surface immobilised probes. The theoretical minimum detectable concentration for sensors where biomolecules are immobilized onto the surface is dependent on the incubation time and the dimensions of the surface. For example, in a time period of 100 s, targets displayed on two-dimensional surfaces are limited to 0.1 picomolar range, with femtomolar detection requiring incubation periods of hours to days [59]. As environmental monitoring requires sub-femtomolar detection and as contaminants in environmental samples can lead to rapid degradation of environmental DNA [60], surface-bound sensors will not be appropriate for environmental AMR monitoring unless they are coupled with an appropriate system to counter this limitation of biomolecule diffusion rates such as integration with microfluidics channels to address diffusion limits of biomolecules from the solution to the sensor. Such systems have already been developed [61,62].

### 3.3. Combined Methods

Methods combining DNA amplification and detection of the amplicons by surface hybridisation combine the high sensitivity generally attributed to amplification techniques and the added selectivity of both methods. Moreover, such double sieve methods allow spatial separation of the signals generated by multiple targets at one time, which is not possible with amplification methods alone. During the first step, a specific region of the gene of interest is amplified and labelled using specific primers, increasing the ratio of target DNA to unspecific background DNA. Alternatively, if the DNA complexity is low and DNA level is limiting, technology is now available to amplify entire genomes uniformly and without bias, for example the GenomePlex^®^ Single Cell Whole Genome Amplification Kit. In a second step, the microarray or hybridization probe specifically binds to and detects the DNA biomarker of interest, bypassing any unspecific DNA sequence that may have been amplified in the first step. In addition to increasing specificity, the labelling of the analyte is carried out during the amplification phase which circumvents the drawback associated with sample labelling with surface bound hybridisation. Furthermore, increasing the sample pool removes the requirement of integrating the sensor element with microfluidics channels to address diffusion limits of biomolecules and could increase target levels to those compatible with quantification by optical transduction systems, which are low cost and amenable to automation. However, the use of such hybrid sensing technologies retains the disadvantages of DNA amplification techniques described in the section above.

### 3.4. Sample Preparation Technology

**Figure 3:**
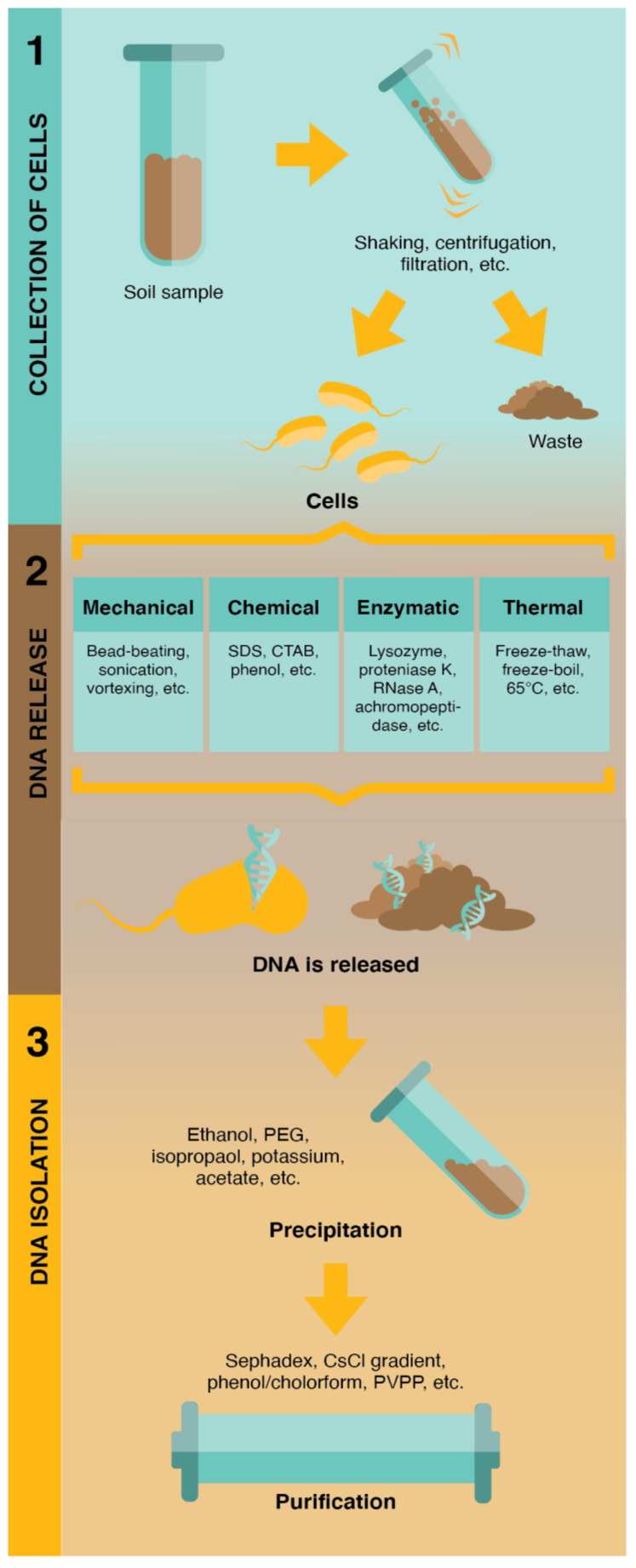
The three steps of environmental DNA extraction: 1. isolation of the cell fraction, 2. cell lysis and DNA release, 3. DNA isolation.

Any sensing technology will require extraction of the DNA from the environmental sample into a medium suitable for the sensor. Despite a staggering diversity in the technical details, the literature addressing the extraction of DNA from environmental samples adheres to the same general three-step design (Figure 3): collection of the cell fraction (an optional step); DNA release; DNA extraction and DNA isolation. Developments in this area have been primarily motivated by specific ecology and biodiversity research, with protocols developed accordingly.

Environmental samples contain both extracellular and intracellular DNA. Generally extracellular DNA will be partially decomposed and bound to inorganic particles or other non-biological debris and can be selectively extracted with relative ease. Intracellular DNA is protected within a cell and consequently is relatively pristine. If extracellular DNA alone is required then the first two steps of the process outlined in Figure 3 are skipped; if both extracellular and intracellular DNA are required then the first step is skipped, otherwise the process is as outlined below.

Given that sensitivity and selectivity of any method is dependent on the length of hybridisation between probe and target, it is likely that environmental sensing will require use of intracellular DNA. Initially, the microbial cell fraction may be collected from the bulk sample by physical methods such as centrifugation or filtration resulting in high quality DNA, but at low yield [63]. Cell lysis and DNA extraction is then performed [64]. This process involves releasing DNA from sediment and cells based on various treatments: chemical, thermal, enzymatic, or mechanical. If the internal cell fraction has not been specifically collected in step 1, then the extract from this method will contain both intra- and extracellular DNA components, with the length and quality of DNA dependent on the method used. There is a wide variety of reagents and process hardware that can be used and it is common practice to create a hybrid approach combining two or more techniques. For example, the custom DNA extraction kit, GnS-GII developed for large-scale soil surveys [65], involves a combination of bead-beating (FastPrep, MP Biomedical) and detergent (SDS) for lytic DNA release. The Sy3 procedure, optimized for environmental samples with low cell abundances [66,67], uses all four techniques: bead-beating (Mikrodismembrator, B. Braun Biotech), detergent (SDS), enzymatic digestion (proteinase K), and freeze–thaw cycles.

The final step is isolation of the DNA in preparation for analysis. Here, the literature surrounding environmental DNA isolation is extremely biased towards PCR analysis. Environmental samples contain materials that are incompatible with PCR analysis, and extraction protocols involve the removal of materials that can interfere with the analysis such as heavy metal salts, phenolic polymers, DNA-binding proteins and other cell debris. The most scrupulous measures are those employing chromatography or gel electrophoresis, or combinations of the two [68,69]. Under less stringent requirements, the DNA can simply be precipitated, and recovered from a filter membrane or silica beads [70].

To date, the final results obtained from studies from DNA from the environment are significantly influenced by the chosen steps within the DNA extraction protocol [71,72,73,74,75,76]. Extraction of DNA from cells is arduous and leads to species bias when DNA from easy-to-lyse cells is preferentially represented compared to DNA from more robust cells. Table 1 contains multiple examples of studies documenting bias as a result of DNA extraction protocols. The effects include expected variations in DNA yield and quality. However, more surprising consequences include disagreement over the relative abundances of microorganisms within a sample.

Significant efforts have focused on developing unbiased general procedures [77,78,79,80,81,82,83,84,85,86], and associated devices [87,88]. The International Organization for Standardization has developed a standard (ISO standard 11063) for DNA extraction and analysis [89,90,91,92], that has been revised in 2020, to facilitate the comparison of results from geographically widespread measurements across continents and borders. ISO 11063 is mostly dedicated to agricultural and forest soils and its appropriateness for AMR monitoring in other environments still needs to be determined. In reality, it is unlikely that totally unbiased methods that are economical and suited to automated or point of use devices will be developed. More critical is development of a standard method, with known bias, that can be used broadly for surveillance.

## 4. Conclusions and Outlook

If environmental AMR surveillance systems are to be geographically and temporally widespread, they will require point-of-use, or autonomous sensing devices. Current AST methods to detect AMR in the clinic, that are primarily designed to provide treatment guidance to clinicians, are not ideal for surveillance aimed at providing insight into AMR in the broader environmental bacterial ecosystem. Cheaper and more easily deployable devices that quantify DNA sequences which act as biomarkers of AMR are more likely to achieve a widespread surveillance system to monitor levels of ARGs in the environment than methods based on phenotypic assessment.

Devices based on DNA detection will need to match sensitivity and selectivity specifications of AMR biomarkers found in the environment. At this point in time, no technique can detect DNA across the dynamic range of ARGs found in the environment. Hence, there is a requirement to adjust the concentration of samples to the dynamic range of any detection method prior to detection. Technologies based on DNA hybridisation are by far the most sensitive methods available for detecting DNA. The sensitivity of these methods is based on the DNA-DNA hybridisation event and signal transduction methods. To date most effort has focused on the signal transduction component. Advances in sensitivity and selectivity may arise from basing technology on longer regions of DNA-DNA hybridisation of at least 50 nucleotides.

Methods with potential to fulfil these criteria include DNA amplification-based methods, and surface immobilized DNA coupled methods that detect surface changes post hybridization. However, substantial challenges will need to be addressed for both types of technologies. In addition, these methods will require integration with disperse and collect strategies, e.g., microfluidic channels to address diffusion limits of biomolecules from solution to the sensor surface.

Precise details of the methods to extract DNA from environmental samples will be highly dependent on the sensor technology but will likely follow a standard three-step design: collection of the cell fraction, DNA release and DNA isolation. It is clear that extraction protocols strongly bias results and that standardized methods are required to allow comparison of geographically widespread measurements across borders.

## Figures and Tables

**Table 1:** Studies documenting biases introduced by various DNA extraction protocols. This table is an extended version from Mbareche et al. (2017) [76].

Target	Method	Findings	Reference
Bacteria/ eukaryote/ archea	Metagenomics	Abundance of microbes varied significantly with DNA extraction methods.	[93]
Bacteria	Metagenomics; PCR-based sequencing	Bacterial DNA contamination in commercial DNA extraction kits obstructed analysis.	[94]
Virus	Quantitative PCR	Extraction protocol affected recovery yields.	[95]
Fungi	Quantitative PCR	Extraction protocol affected recovery yields.	[96]
Bacteria	PCR-based sequencing	Lysis method biased estimation of certain phyla.	[97]
Bacteria	PCR-based sequencing	Lysis method biased estimation of bacterial distribution.	[98]
Bacteria/fungi/ archea	Quantitative PCR	Ten extraction kits generated significantly divergent results.	[99]
Viruses	Quantitative PCR	Extraction protocol affected recovery yields.	[100]
Bacteria	PCR-based sequencing	Bacteria abundance varied significantly with DNA extraction method.	[101]
Bacteria	PCR-based sequencing	Bacterial DNA contamination in commercial DNA extraction kits obstructed analysis.	[102]
Bacteria/archea	PCR-based sequencing	Microbial abundane varied significantly with DNA extraction method.	[103]
Bacteria	Quantitative PCR	Bead sizes used in lysis step affected DNA yields	[104]
Bacteria	Metagenomics + PCR-based sequencing	Eight extraction kits generated significantly divergent results.	[105]
Eukaryota	Metagenomics + PCR-based sequencing	Three extraction kits generated significantly divergent results.	[106]
Eukaryota	PCR-based sequencing + quantitative PCR	Three extraction kits generated significantly divergent results.	[107]
Bacteria	Metagenomics	Five extraction kits generated significantly divergent results.	[108]
Bacteria	Metagenomics + quantitative PCR	Two extraction kits generated significantly divergent results.	[109]
Fungi	PCR-based sequencing + quantitative PCR	Three extraction kits generated significantly divergent results.	[110]
Bacteria	PCR-based sequencing	Lysis protocol biased results.	[111]
All	Metagenomics	Comment on the delusion of an all-purpose environmental DNA extraction technique.	[73]
Bacteria	DGGE profiling	Four in situ lysing protocols produced significantly divergent results.	[112]
Bacteria	DGGE profiling	Extraction and purification protocols affected yield and average molecular weight of extracted DNA.	[113]
Bacteria	DGGE profiling	Extraction and purification protocol affected yield and average molecular weight of extracted DNA.	[114]
Bacteria	Blotting; DGGE profiling	Lysis protocol biased Gram-negative and positive bacteria.	[115]
